# Fetal Cerebellar Area: Ultrasound Reference Ranges at 13–39 Weeks of Gestation

**DOI:** 10.3390/jcm12124080

**Published:** 2023-06-16

**Authors:** Luigi Manzo, Giuliana Orlandi, Olimpia Gabrielli, Paolo Toscano, Enrica Di Lella, Antonia Lettieri, Laura Letizia Mazzarelli, Giordana Sica, Letizia Di Meglio, Lavinia Di Meglio, Gabriele Ruffo, Carmine Sica, Ferdinando Antonio Gulino, Giosuè Giordano Incognito, Attilio Tuscano, Alice Giorno, Aniello Di Meglio

**Affiliations:** 1 Department of Neuroscience, Reproductive Sciences and Dentistry, School of Medicine, University of Naples Federico II, 80138 Naples, Italy; luigimanzo93@libero.it (L.M.); giulianaorlandi@msn.com (G.O.); olimpia.gabrielli3@gmail.com (O.G.); paol.toscano@gmail.com (P.T.); enrica_dilella@hotmail.it (E.D.L.); lauramazzarelli@gmail.com (L.L.M.); giorno.alice@libero.it (A.G.); 2 Diagnostica Ecografica e Prenatale di A. Di Meglio, 80133 Naples, Italy; antonia_lettieri@libero.it (A.L.); gabrieleruffo89@libero.it (G.R.); sicacarmine111@gmail.com (C.S.); aniellodimeglio@gmail.com (A.D.M.); 3 School of Medicine, University of Campania Luigi Vanvitelli, 81031 Caserta, Italy; giordanasica@icloud.com; 4 Radiology Department, School of Medicine, University of Milan, 20122 Milan, Italy; letiziadimeglio@gmail.com; 5 Pediatric Department, Bambino Gesù Children’s Research Hospital IRCCS, 00165 Rome, Italy; laviniadimeglio@gmail.com; 6 Department of Obstetrics and Gynaecology, Azienda di Rilievo Nazionale e di Alta Specializzazione (ARNAS) Garibaldi Nesima, 95124 Catania, Italy; 7 Department of General Surgery and Medical Surgical Specialties, University of Catania, 95123 Catania, Italy; giordanoincognito@gmail.com (G.G.I.); attiliotuscano@gmail.com (A.T.)

**Keywords:** ultrasound, cerebellum, posterior fossa anomalies, biometry

## Abstract

Background and Objectives: The present study aims to provide prenatal 2-dimensional ultrasonographic (2D-US) nomograms of the normal cerebellar area. Materials and Methods: This is a prospective cross-sectional analysis of 252 normal singleton pregnancies, ranging from 13 to 39 weeks of gestation. The operator performed measurements of the fetal cerebellar area in the transverse plane using 2D-US. The relationship between cerebellar area and gestational age (GA) was determined through regression equations. Results: A significant, strong positive correlation was investigated between the cerebellar area with GA (r-value = 0.89), and a positive correlation indicates that with increasing GA, the cerebellar area increased in all the participants of the study. Several 2D-US nomograms of the normal cerebellar area were provided, and an increase of 0.4% in the cerebellar area each week of GA was reported. Conclusions: We presented information on the typical dimensions of the fetal cerebellar area throughout gestation. In future studies, it could be evaluated how the cerebellar area changes with cerebellar abnormalities. It should be established if calculating the cerebellar area in addition to the routine transverse cerebellar diameter may help in discriminating posterior fossa anomalies or even help to identify anomalies that would otherwise remain undetected.

## 1. Introduction

Without a shadow of a doubt, the realm of obstetric practice presents a complex and multifaceted landscape that ceaselessly evokes fascination and curiosity. Undeniably, at the pulsating heart of this captivating medical discipline, one critical element shines through its pivotal significance—the methodical, meticulous, and precise assessment of the fetal development process. A task of immense gravity and importance, this particular duty is far from being a static or unchanging procedure; instead, it can be more appropriately characterized as a vibrant and dynamic process that constantly evolves, changes, and adapts in line with the unstoppable forward march of technology. In a world where advancements in technology incessantly bring forth new tools, techniques, and methodologies, our understanding of fetal development expands correspondingly. These technological leaps not only deepen our comprehension of the intricate process but also push the boundaries of our explorative capabilities, allowing us to engage in a more thorough, detailed, and comprehensive assessment than ever thought possible in yesteryear. The study of the brain is an important aspect and is based on the definition of its dimensions and the morphology of its components [1]. Among the structures, the assessment of the cerebellum is increasingly debated and evolving [1,2]. Situated within the confines of the posterior cranial fossa, the cerebellum is a key player in the architecture of the hindbrain, boasting the status of being its largest component. It consists of a central part known as the vermis and two convex lateral expansions termed the cerebellar hemispheres. Its external surface has a complicated network of fissures that delimit the flocconodular, anterior, and posterior lobes. Each of these lobes can be further divided into smaller subunits called lobules, the presence of which serves to significantly increase the overall surface area of the cerebellum, thereby enhancing its functional efficiency and capacity. Renowned for its crucial role in controlling and coordinating movement, the cerebellum’s capabilities extend much further. In addition to motor control, it profoundly influences a variety of cognitive functions, including but not limited to attention, memory processing, and language comprehension. Furthermore, it plays an instrumental role in regulating emotions, with a particular emphasis on fear and pleasure. The cerebellum is one of the earliest brain structures to differentiate during the embryonic stage, yet it is also one of the last to reach full maturity. This prolonged developmental timeline, spanning both embryonic stages and the postnatal period, leaves the cerebellum vulnerable to a range of potential developmental anomalies. Brain development begins with a fundamental biological process known as neurulation. This process involves the transformation of the neural plate, formed from the ectodermal layer, into encephalic vesicles at its cephalic end, with the remaining part of the neural plate laying the groundwork for the formation of the spinal cord [3]. In the early stages, the brain architecture comprises three primary encephalic vesicles, called the forebrain, the midbrain, and the rhombencephalon. As development advances, these vesicles undergo further diversification. The forebrain splits into two additional vesicles, namely the telencephalon and the diencephalon, while the rhombencephalon divides into the metencephalon and the myelencephalon. The development of the cerebellum involves the alar plate, which is the dorsal part of the metencephalon, and the neural folds, the future rhombic lips. The alar plate, during its lateral expansion, gives birth to structures called rhombomeres. These rhombomeres then undergo a process of medial fusion, which defines the cavity of the fourth ventricle, leading to the formation of a smooth, convex structure known as the rudiment of the cerebellum. Included within this rudiment is the midline vermis, a critical component that plays a vital role in the cerebellum’s operation [1]. The cerebellar fissures, initially appearing on the surface of the vermis and the floccular region during the fourth month of development, extend to the level of the hemispheres from the fifth month onward [1,4,5,6]. After the 19th week of gestation, the mass of the cerebellum undergoes a substantial increase, doubling in size. This remarkable growth continues beyond birth into the postnatal period [1,7,8,9,10]. Consequently, the evolution of the cerebellum can provide invaluable insights into the fetus’s overall developmental progress [1].

Researchers have conducted numerous studies to understand normal cerebellar growth patterns and establish nomograms of cerebellar dimensions to aid in prenatal diagnosis. For example, Rizzo et al. [11] established reference limits for the cerebellar vermis using three-dimensional ultrasonography images. Alpay et al. [12] constructed nomograms for brainstem structures using two-dimensional ultrasonography (2D-US). A study by Chang et al. [13] demonstrated the effectiveness of three-dimensional ultrasound in assessing fetal cerebellar volume throughout normal gestation, leading to the development of volume-based nomograms. A multitude of ultrasound studies have underscored the clinical significance of transverse cerebellar diameter (TCD) and vermis dimension measurements. These have been proposed as potential alternatives to determining gestational age (GA), providing a fresh perspective compared to the traditional method of measuring the biparietal diameter (BPD) [1,14,15,16,17]. Despite the potential clinical significance of these methods in terms of both indicating normal brain development and potentially detecting brain abnormalities, the prenatal ultrasound evaluation of the cerebellar area has yet to be fully explored and understood.

The present study aims to measure the cerebellar area in fetuses exhibiting normal development by utilizing prenatal 2D-US examinations in the transverse plane throughout the gestational period and to provide 2D-US nomograms of the cerebellar area, creating a potentially invaluable tool that could substantially aid future prenatal assessments.

## 2. Materials and Methods

The current study was designed as a prospective cross-sectional investigation aimed at providing a comprehensive analysis of the intricate relationship between the cerebellar area and GA in a selected cohort of pregnant women. The inclusion criteria included the requirement of a well-documented last menstrual period, serving as an essential reference point for estimating GA. Additionally, crown-rump length (CRL) measurements obtained during first-trimester ultrasound examinations were employed to confirm and accurately determine GA. By employing these standardized methods, the study aimed to establish a solid foundation for accurate GA estimation, which is crucial for investigating the relationship between cerebellar development and GA. Furthermore, fetuses were singleton and non-anomalous to eliminate potential confounding factors associated with multiple gestations. This approach allowed for a more focused investigation into the specific impact of GA on cerebellar development, as it minimized potential variations resulting from differences in the number of fetuses or abnormalities. Moreover, the study considered only pregnancies with the estimated fetal weight falling within the 10th to 90th percentile range, aiming to capture a representative sample of the general population and avoiding any bias towards extreme fetal growth patterns. Moreover, the included participants had negative histories of systemic diseases, normal amniotic fluid volume, intact fetal membranes, and were not in labor at the time of enrollment.

The evaluation of the cerebellar area was conducted during routine ultrasound examinations, performed for first-, second-, and third-trimester screening. This multidimensional approach allowed for a comprehensive analysis of cerebellar development throughout pregnancy, capturing the dynamic changes that occur across different periods.

To ensure consistency and facilitate meaningful comparisons, a standardized approach was adopted for categorizing GA. Fractions of weeks were rounded to the nearest whole week, providing a uniform system for characterizing GA across the study population. Weeks with a GA of ≤4 days were assigned to the lower week, while weeks with >5 days were assigned to the higher week. This systematic categorization ensured the accurate representation of GA and allowed for the exploration of cerebellar development at various stages of pregnancy.

Only healthy neonates with no evidence of growth disturbances (such as growth restriction or macrosomia) were included. This approach aimed to establish a baseline understanding of cerebellar development in the absence of significant deviations from the norm. Adhering to the principles of cross-sectional studies, each fetus was included only once to ensure the independence of data points and prevent potential duplication, further strengthening the study’s validity and reliability.

**Figure 1:**
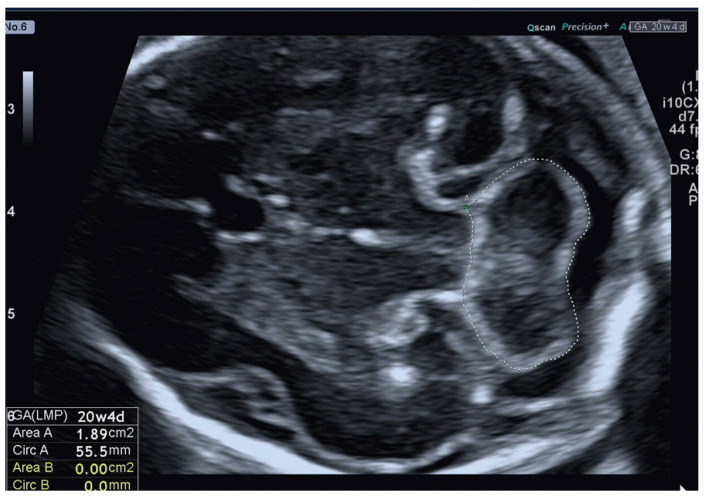
Assessment of the fetal cerebellar area by 2D-US transabdominal approach.

The 2D-US examinations were performed using standard Aloka (Aloka Co., Ltd., Tokyo, Japan) and Voluson E10 (GE Healthcare Ultrasound, Milwaukee, WI, USA) machines equipped with a curved linear array transabdominal transducer (2–5 MHz) and a transvaginal 4–8 MHz probe. The fetal cerebellum was assessed in the transverse plane of the fetal brain, including the cavum septum pellucidum, cerebellum, and cisterna magna, during fetal and maternal rest using a transabdominal acquisition angle of 45–60°, depending on GA. By focusing on this specific plane, the study ensured a standardized approach and consistent measurement technique across all participants. Freeze-frame capabilities allowed for the capture of static images at specific moments, enabling detailed analysis and precise measurements of the cerebellar area. Additionally, an electronic on-screen manual trace was employed to outline the boundaries of the cerebellar structures (Figure 1).

The decision was made not to incorporate color Doppler imaging into the examination protocol. By excluding the color Doppler, the focus remained solely on measuring the cerebellar area without the potential influence of vascular dynamics. This approach ensured that the measured cerebellar area was specific to the cerebellar structures of interest and minimized potential confounding factors related to blood flow patterns.

The cerebellar area was acquired in cross-section with a hand trace, involving a systematic tracing process guided by specific anatomical landmarks. The tracing started at the anterior border of the posterior wall of the spinal cord bridge, followed by the tracing along the contours of the two cerebellar hemispheres. Subsequently, the tracing continued along the posterior margin of the cerebellar vermis, capturing the entirety of the cerebellar area of interest. In cases where the transabdominal route did not provide a clear transverse view of the fetal brain, the transvaginal approach was utilized. It was often due to the fetal position or maternal habitus, which could impact the optimal visualization of the cerebellum. By incorporating this approach, the study aimed to ensure accurate cerebellar measurements, regardless of any potential imaging challenges. The cerebellar area was assessed during the routine scan and obtained by means of two measurements.

The statistical analysis was carried out using GraphPad Prism version 8.4.2 for Windows (GraphPad Software, San Diego, CA, USA) and IBM^®^ SPSS^®^ statistical software version 21.0 (SPSS Inc., Chicago, IL, USA). Only cases with GA between 13 and 39 weeks were included in the analysis. The clinical characteristics of the participants were expressed as the mean and standard deviation. These descriptive statistics provided a comprehensive overview of the central tendency and variability within the study population, offering insights into the overall characteristics of the sample. The clinical characteristics of the participants were expressed as mean and standard deviation, and the normal distribution of the data was assessed using the Kolmogorov-Smirnov test. Spearman’s rank correlation coefficient and scatter plots were drawn to explore the relationship between the cerebellar area and the GA. A linear regression analysis equation was calculated to highlight how the increase in GA accelerated the progression of the cerebellar area. A *p*-value less than 0.05 was considered statistically significant, indicating meaningful associations between variables.

## 3. Results

The study comprised 283 pregnant women who fulfilled the inclusion criteria.

Measurements of the cerebellar area were performed on all 283 fetuses between 13 and 39 weeks of gestation.

The cerebellum appeared as a “butterfly image”, with two symmetrically curved hemispheres conjoined by a hyperechoic structure, known as the cerebellar vermis. There appeared to be no change in its sonolucency between 13 and 39 weeks of gestation.

We obtained satisfactory cerebellar area measurements in the large majority of cases. Precisely, in 89.9% of the instances, which equates to 252 out of the total 283 cases, we considered the measurements to be adequately accurate. We adopted a dual approach in our measurement methodology, which involved either a transabdominal technique, applied in 225 cases, or a transvaginal technique, utilized in 32 cases. The choice of technique was determined by the unique specifics of each case. In a small subset of cases, representing approximately 10.1% of the total cohort, the measurements could not be included in our analysis. In these specific cases, factors such as fetal position and certain attributes of the maternal body structure hindered an optimal evaluation of the fetal cerebellum. More specifically, these factors resulted in our inability to capture a satisfactory transverse plane image, including the cavum septum pellucidum, the cerebellum, and the cisterna magna.

To encapsulate the clinical characteristics of the study population, we compiled these details into Table 1. This compilation provides an account of the Hadlock Ultrasound measurements used in our study.

In Table 2, the predicted 10th, 50th, and 90th percentiles of the cerebellar area, expressed in square centimeters (cm^2^), as a function of the GA, expressed in weeks, were reported.

**Figure 2:**
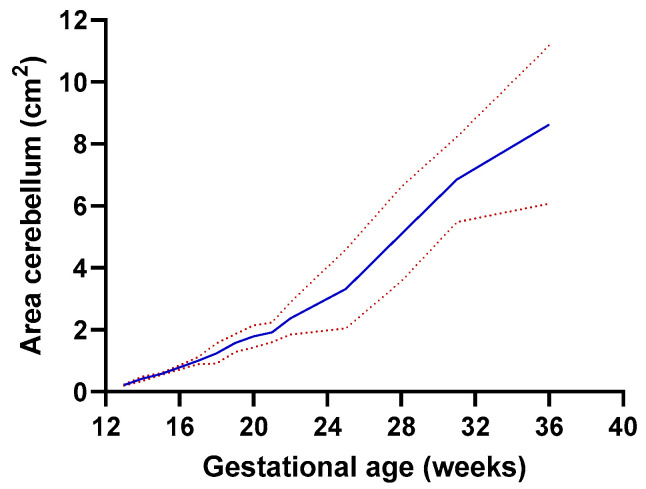
Plot showing the cerebellar area observed measurements and the fitted 10th (lower line), 50th (median line), and 90th (higher line) percentiles for gestational age.

One of the outcomes of our study was the discovery of a positive correlation between GA and the cerebellar area. This correlation suggests that as the GA advances, there is a corresponding increase in the cerebellar area. This finding was consistent across all participants in the study (Table 3) (Figure 2).

Our research conclusions were further reinforced by the regression equations we derived. These equations encapsulate the relationship between the mean cerebellar area (represented by y) and GA (represented by x). This relationship is captured by the equation: y = 0.4176x − 6.692. Additionally, to gain a comprehensive understanding of the data, we derived a second equation, which defines the relationship between the standard deviation of the cerebellar area (represented by y’) and GA: y’ = 0.009x + 0.215. Collectively, these equations suggest a steady increase of approximately 0.4% in the cerebellar area for each week of GA progression.

## 4. Discussion

Cerebellum development occurs over a long period of time, so it has a high susceptibility to experiencing various disorders [18]. One of the most common posterior fossa anomalies is the Chiari malformation. This malformation manifests as an extension of the lower part of the cerebellum into the spinal canal. This can lead to compression of the brain tissue and obstruct the flow of cerebrospinal fluid, leading to potential complications. There are four types of Chiari malformations. Type I is the most common variant, where the lower part of the cerebellum extends into the opening at the base of the skull. This type often does not cause any symptoms and may be discovered incidentally during imaging performed for another reason. Type II, on the other hand, is usually associated with spina bifida, a birth defect where the spinal cord does not develop properly. This type results in both the cerebellum and the brainstem being displaced into the spinal canal, leading to compromised breathing and swallowing functions. Type III is a rare and severe form where the cerebellum and brainstem extend into a sac that protrudes through an opening at the back of the skull. This can result in severe neurological symptoms and is often diagnosed shortly after birth. Finally, type IV is the rarest and most severe of all, where the cerebellum fails to develop properly. This malformation is often fatal before or shortly after birth. In Dandy-Walker malformation (DWM) [19], the cerebellar vermis does not fully develop, resulting in an enlarged posterior fossa and fourth ventricle. This is accompanied by an upward displacement of the lateral sinuses, tentorium, and torcular [19,20]. In this pathology, the TCD can be normal. Vermis hypoplasia is a cerebellar malformation difficult to diagnose prenatally due to the normal vermis position or minimal upward rotation (without tentorium elevation). The fluid collection behind the cerebellum is typically minor and is directly connected to the fourth ventricle [21]. Another category of cerebellar malformations includes cerebellar hypoplasia, characterized by a reduction in cerebellar volume. This group of disorders presents heterogeneity in its manifestations, and the TCD is usually small when measured in the axial or coronal plane. The diagnosis of cerebellar hypoplasia typically occurs late in pregnancy or after delivery due to its late onset and the fact that the TCD is not routinely evaluated during the third trimester [19]. Among the rare cerebellar malformations is pontocerebellar hypoplasia (PCH), which is characterized by the prenatal onset of cerebellar hypoplasia with superimposed atrophy [19]. Fetuses with PCH are very infrequently diagnosed. According to Leibovitz et al. [22], there were only two cases of PCH diagnosed at 29 gestational weeks, and both cases exhibited reduced midbrain and hindbrain measurements. The authors concluded that relying solely on prenatal imaging for the diagnosis of this disorder could prove unreliable. Another rare congenital cerebellar defect is rhombencephalosynapsis (RES), characterized by a complete or partial absence of the vermis along with fused cerebellar hemispheres, middle cerebellar peduncles, and dentate nuclei [23]. Usually, the transcerebellar diameter in these cases is smaller than average. The typical fissure between the hemispheres is indistinguishable on the axial plane, and the dorsal part of the cerebellum displays a circular shape and does not show the typical “butterfly” shape; transverse cerebellar folias appear continuous and cross the midline. In the sagittal view of the brain, the fourth ventricle takes on a circular shape, and the main fissure is imperceptible [19]. The detection of RES using ultrasound before 22 weeks of gestation is infrequent, and patients are typically referred for fetal ventriculomegaly [24,25]. Generally, the TCD in such cases is smaller than expected. Joubert syndrome is a rare autosomal recessive genetic disease associated with syndromic retinitis pigmentosa and characterized by the absence or underdevelopment of the cerebellar vermis and a brainstem malformation that gives a typical “molar tooth” appearance [19,26]. A prenatal diagnosis is extremely difficult in such cases. On the transcerebellar plane, the vermis appears hypoplastic, missing the inferior part and producing a midline cleft connecting the 4th ventricle to the cisterna magna [27]. Prenatal diagnosis has been described following a positive family history or when associated with other typical anomalies, such as renal malformations [28]. The visualization of the molar tooth is difficult and requires ultrasound scans performed by experienced operators. Axial magnetic resonance imaging (MRI) scans usually aid in the diagnosis by allowing the visualization of the pathognomonic “molar tooth sign” [27,29].

Ultrasound serves as an essential diagnostic instrument for a multitude of obstetric and gynecological conditions [30,31,32,33,34,35]. A crucial application of this technology is the examination of the fetal brain. In particular, the evaluation of the fetal posterior fossa using ultrasound typically involves visualizing axial planes, including coronal and sagittal planes, via the transabdominal approach. The axial plane enables the assessment of the vermis and the cerebellar hemispheres, the TCD, the fourth ventricle size, and the cerebellar peduncle thickness [19]. TDC measurement is part of the second-trimester routine scan [36] and part of the assessment of the fetal brain [37]. This involves measuring the cerebellar diameter in the transcerebellar plane, which is a plane passing through the thalamus and cavum septum pellucidum. In this section, it is possible to identify the occipital horns of the lateral ventricles, the thalami, the interhemispheric fissure, and the cerebellum [38], which, in cross-section, appears as a butterfly-shaped structure, with the vermis recognizable as being slightly more echogenic than the two cerebellar hemispheres [27]. Among the coronal planes, the only one that can be acquired via the posterior fontanelle is the transcerebellar plane [37]. The vermis can also be measured in the same plane as TDC. The length, width, and thickness of the vermis can be measured to assess its size and growth. There is a lot of research about the TDC, correct vermis measurement, and nomograms to use during the ultrasound for each GA. For example, at 20 weeks of gestation, the average TCD measurement range is between 16.3 mm and 22.1 mm, and at 32 weeks of gestation, the average TCD measurement range is between 31.1 mm and 40.7 mm. These ranges can vary slightly depending on the specific nomogram used in the measurement. [19,22,39,40].

Yet, there are no reports comparing the cerebellar area to nomograms to define abnormal growth. Therefore, we suggest that the cerebellar area might be a useful parameter for its actual size. We provided prenatal 2D-US nomograms of the normal cerebellar area and reported an increase of 0.4% in the cerebellar area each week of GA.

Moreover, both TDC and vermis measurements can be used to detect a range of fetal abnormalities. Posterior fossa anomalies can be suspected during the first-trimester screening ultrasound, but they must be confirmed in a second-trimester scan [19]. Anomalies during the second and third trimesters are typically detected when a small cerebellum, morphological anomalies, or communication between the fourth ventricle and the cisterna magna are visualized during routine screening [19,41]. Although most of the cerebellar anomalies can present with a reduced TCD, any of these can show only a morphological anomaly in association with a normal TDC. In these cases, an untrained operator may fail in its recognition, and using the cerebellar area assessment for biometric evaluation could be supportive in the differential diagnosis of posterior fossa abnormalities or even help identify abnormalities. The transcerebellar plane is the landmark also used for the measurement of the cerebellar area. The technique used to measure the cerebellar area is the hand trace, following anteriorly the posterior wall of the spinal cord bridge, continuing along the two cerebellar hemispheres, and finally passing posteriorly along the posterior margin of the cerebellar vermis. Using the cerebellar area measurement may be helpful to suspect the presence of cerebellar and posterior fossa anomalies and refer the patient to a second-third level center if further studies are necessary.

While TCD measurement can be easily evaluated by sonographers, the morphological assessment of the cerebellum may be challenging for less trained operators. We could not obtain the cerebellar area measurement in 10.1% of cases because an adequate transverse plane could not be obtained due to the fetal position or the maternal habitus. However, the transcerebellar plane is the same one used for the measurement of both TDC and vermis measurements, whose evaluations would be inadequate in the same percentage of cases as the cerebellar area.

A limitation of the present study is the small population sample. Larger population studies will be necessary to demonstrate the feasibility of this measurement. Moreover, the data about the first-trimester cerebellar area are limited, and more studies are necessary. The TCD measurement has not been reported, and it would be useful to compare the cerebellar area to this already known and used parameter in future research.

## 5. Conclusions

We presented information on the typical dimensions of the fetal cerebellar area throughout gestation, providing normograms and reporting an increase of 0.4% in the cerebellar area each week of GA. In future studies, it could be evaluated how the cerebellar area changes with cerebellar abnormalities. It should be established if calculating the cerebellar area in addition to the routine TCD may help in discriminating posterior fossa anomalies or even help to identify anomalies that would otherwise remain undetected. In this study, we presented information regarding the typical dimensions of the fetal cerebellar area at various stages of gestation. We constructed nomograms that illustrate the expected growth patterns of the cerebellar area throughout the course of pregnancy, providing healthcare professionals with a valuable reference tool. Moreover, our findings revealed a consistent and remarkable increase of 0.4% in the cerebellar area per week of GA. In order to further advance our knowledge in this field, it is imperative to conduct future research endeavors focusing on comprehensively evaluating how the cerebellar area changes in the presence of various cerebellar abnormalities. By systematically examining and quantifying these abnormalities, we can gain valuable insights into their impact on cerebellar growth patterns and determine whether calculating the cerebellar area, in addition to routine TCD measurements, can serve as an indispensable diagnostic modality for discriminating and precisely characterizing posterior fossa anomalies. The potential clinical implications of integrating cerebellar area calculations into routine screening protocols are highly promising. By augmenting the existing diagnostic arsenal, we may be able to effectively identify and categorize anomalies that might otherwise elude detection. Given the complex nature of cerebellar development and the diverse spectrum of cerebellar abnormalities, we advocate for further investigations in this domain to unravel the intricate mechanisms underpinning cerebellar growth and to delineate the diagnostic accuracy of incorporating cerebellar area measurements in clinical practice.

## Figures and Tables

**Table 1:** Clinical characteristics of the study population using Hadlock Ultrasound measurements.

Characteristics	Value
BPD (mm)	54.90 ± 16.42
BPD (percentile)	55.53 ± 27.17
HC (mm)	204.7 ± 60.89
HC (percentile)	59.84 ± 25.61
AC (mm)	184 ± 60.33
AC (percentile)	60.80 ± 24.43
FL (mm)	39.52 ± 14.32
FL (percentile)	61.02 ± 22.51
EFW (g)	778.4 ± 802.1

AC, abdominal circumference; BPD, biparietal diameter; EFW, estimated fetus weight; FL, femur length; HC, head circumference. Data are presented as mean and standard deviation.

**Table 2:** Predicted 10th, 50th, and 90th percentiles of the cerebellar area (cm^2^) by gestational age (weeks).

Gestational Age	Number of Cases	10th Percentile	50th Percentile	90th Percentile
13	2	0.2	0.22	0.24
14	4	0.35	0.435	0.49
15–16	2	0.55	0.575	0.6
17	3	0.87	1.06	1.07
18	5	0.95	1.16	1.6
19	7	1.3	1.54	1.88
20	48	1.444	1.775	2.162
21	57	1.618	1.89	2.262
22–24	50	1.893	2.265	2.939
25–27	8	1.95	3.57	4.46
28–30	14	3.61	5.005	6.655
31–33	24	5.4	7.04	8.135
34–39	24	6.105	8.545	11.21

**Table 3:** Correlation between the cerebellar area with gestation age.

	Gestation Age (r-Value)	*p*-Value
Cerebellar area (cm^2^)	0.89	<0.0001

r-value, correlation value; Spearman’s Rank correlation test.

## Data Availability

Data are unavailable due to privacy restrictions.
